# *BRAF* V600 mutations in Langerhans cell histiocytosis with a simple and unique assay

**DOI:** 10.1186/s13000-016-0489-z

**Published:** 2016-04-19

**Authors:** Michiko Tatsuno, Yoko Shioda, Hideto Iwafuchi, Shigeki Yamazaki, Kenta Iijima, Chiaki Takahashi, Hiromi Ono, Kiyono Uchida, Osamu Okamura, Mamoru Matubayashi, Torayuki Okuyama, Kimikazu Matsumoto, Takako Yoshioka, Atsuko Nakazawa

**Affiliations:** Department of Pathology, National Center for Child Health and Development, PO Box 157-8535, 2-10-1 Okura, Setagaya-ku Tokyo, Japan; Department of Children’s Cancer Center, National Center for Child Health and Development, PO Box 157-8535, 2-10-1 Okura, Setagaya-ku Tokyo, Japan; Department of Clinical Laboratory Medicine, National Center for Child Health and Development, PO Box 157-8535, 2-10-1 Okura, Setagaya-ku Tokyo, Japan; Department of Pathology, Tokai University School of Medicine, PO Box 259-1193, 143 Shimokasuya, Isehara-shi, Kanagawa Japan

**Keywords:** Langerhans cell histiiocytosis (LCH), V-raf murine sarcoma viral oncogene homolog B1 (BRAF), Formalin-fixed paraffin-embedded (FFPE), TspRI

## Abstract

**Background:**

BRAF (V-raf murine sarcoma viral oncogene homolog B1) is a serine-threonine protein kinase involved in cell survival, proliferation, and differentiation. The most common missense mutation of *BRAF* (mainly V600E) contributes to the incidence of various cancers, including Langerhans cell histiocytosis (LCH). BRAF inhibitors molecularly targeting the V600E mutation have been developed to counteract the effect of the mutation. To ensure the administration of effective pharmacotherapy, it is therefore imperative to develop an effective assay to screen LCH patients for the V600E mutation. However, tumor tissues of LCH typically contain many inflammatory cells which make a correct judgement of the mutation status difficult in the DNA sequence analysis.

**Results:**

In this study, we present a new, highly sensitive analyzing method combining PCR, restriction enzyme digestion, and a sequencing assay using DNA extracted from formalin-fixed paraffin-embedded (FFPE) tissue specimens. TspRI is a restriction enzyme that cleaves the sequence encompassing the wild-type *BRAF* codon 600 into two fragments, which cannot be used as a template for subsequent BRAF PCR amplification. We therefore evaluated the sensitivity of *BRAF* V600 mutation detection by amplifying the primary PCR product digested with TspRI and sequencing the secondary PCR products. The V600E mutation was detected in FFPE tissue samples from 32 LCH patients; our assay was able to identify mutations in four samples that gave inconclusive results, and ten that were negative, according to standard PCR and sequencing.

**Conclusions:**

We presented a new and highly sensitive method to detect *BRAF *V600 mutations. This screening method is expected to play an important role to select the most effective therapies.

## Background

V-raf murine sarcoma viral oncogene homolog B1 (BRAF) is a serine-threonine protein kinase that functions through the RAS/MAPK (RAS-RAF-MEK-ERK) [1] signaling cascade, regulating cell survival, proliferation, and differentiation. Missense mutations in the *BRAF* gene contribute to the incidence of various types of cancer [2, 3]. The V600 mutations account for the majority of *BRAF* mutations and are observed in Langerhans cell histiocytosis (LCH) [4], Erdheim-Chester disease (ECD) [5], melanoma [6], papillary thyroid carcinoma [7, 8], colorectal cancer [9], hairy cell leukemia (HCL) [10], and chronic lymphocytic leukemia (CLL) [11].

Recently, *BRAF* mutations were shown to interfere with pharmacotherapies targeting the epithelial growth factor receptor (EGFR) [12, 13]. Accordingly, BRAF inhibitors targeting the V600 mutations were developed to preserve EGFR responses in melanoma, and they are anticipated to be effective in various cancers associated with *BRAF* V600 mutations. Therefore, it is imperative to develop a sensitive screening method for the detection of V600 mutations to determine which patients may require V600 inhibitors and to ensure the efficiency of EGFR-targeted therapy.

In this study, we present a highly sensitive assay using a combination of PCR, restriction enzyme cleavage, and a sequence analysis of DNA extracted from formalin-fixed paraffin-embedded (FFPE) sections. The sensitivity of the assay was determined by inspecting several samples derived from mixtures of two cell lines, one with the *BRAF* V600E mutation and another with wild-type *BRAF*.In addition, the results using this new method were compared with those from standard PCR and sequencing, and the two methods were evaluated using FFPE tissue section from LCH patients, the tumor content ratio determined by CD1a immunostaining.

## Methods

### Subjects and samples

This study was conducted using FFPE tissue from LCH patients diagnosed at the National Center for Child Health and Development. There were 32 patients in total, 16 males and 16 females. Twenty-three patients were aged ≤5 years and nine were aged ≥6 years. The FFPE samples were from the bone or tissue (*n* = 14), or subcutaneous tissues (*n* = 18). Normal control DNA was extracted from a human tonsil FFPE block. This study was approved by the Ethics Committee of the National Center of Child Health and Development (No. 559, 1035), and patients (or their guardians) provided their written informed consent for the use of their samples in this study.

### Cell lines

The *BRAF* V600E mutant cell line, A2058, and the wild-type cell line, UE7T-13, were used to verify the efficiency of *BRAF* mutation detection. A2058 and UE7T-13 were acquired from the JCRB Cell Bank (National Institute of Biomedical Innovation). Cells were cultured in DMEM (GIBCO: catalogue number 12430-054) containing 10 % FCS (Sigma-Aldrich).

Six mixtures were prepared by combining various proportions (0, 5, 10, 20, 50 and 100 %) of the A2058 *BRAF* V600E mutation (+) cell line with the UE7T-13 BRAF mutation (-) cell line; FFPE cell blocks were prepared for each mixture using the Shandon Cytoblock kit (Thermo Scientific), and 5 × 10 μm sections were cut from each block. Three of these five sections were collected in 1.5 ml Eppendorf tubes for DNA extraction and molecular analyses in triplicate.

### DNA extraction

DNA was extracted from the FFPE sections using the ReliaPrep FFPE gDNA Miniprep System (Promega) or the NucleoSpin FFPE DNA Kit (Macherey-Nagel) according to the manufacturer’s protocols. The concentration of the extracted DNA was determined using a NanoDrop spectrophotometer (Thermo Scientific).

### PCR and sequence analysis of *BRAF*

We designed one set of primers to PCR amplify *BRAF* exon 15, including the codon 600 sequence, and generate a product of 209 bp. The forward primer sequence was BRAF-F: 5′-TCATAATGCTTGCTTGCTCTGATAGGA-3′ and the reverse primer was BRAF-R: 5′-CAGTGGAAAAATAGCCTC-3′ (nucleotides 147–169 and 355–338 of GenBank: M95712.2, respectively). PCR was performed with 25 μl reaction mixtures, using the HotStarTaq Master Mix Kit (Qiagen), containing 12.5 μl of 2 × reaction master mix, each primer at a final concentration of 0.4 μM and 1 μl of template. The PCR conditions were as follows: an initial denaturation at 95 °C for 15 min, followed by 42 cycles of amplification (30 s at 95 °C, 40 s at 56 °C, and 40 s at 72 °C), and a final step of 72 °C for 10 min. These PCR conditions were used for first and second PCR. Molecular sizes and concentrations of the PCR products were determined using a Bioanalyzer (Agilent).

The PCR products were treated with ExoSAP-IT (Affymetrix) to remove unconsumed dNTPs and primers. A sequence analysis was subsequently performed according to the BigDye Terminator v3.1 Cycle Sequencing kit protocol (Applied Biosystems) using the forward or reverse primer. Free dye terminators were removed from the completed sequencing reactions using a DyeEx 2.0 Spin kit (Qiagen), followed by ethanol precipitation and resuspension in Hi-Di Formamide (Applied Biosystems). The sequence data were detected using an ABI PRISM 3100 Genetic Analyzer (Applied Biosystems), and the mutation sequence was confirmed by both the forward and reverse sequence data.

### Restriction enzyme analysis

TspRI (New England Biolabs) is a restriction enzyme that cuts at the sequence 5′-NNCASTGNN-3′ (3′-NNGTSACNN-5′); it can cut at the wild-type V600 *BRAF* codon in PCR amplicons across this region to give two fragments, but not sequences with V600E or V600D mutations. Digestion of PCR products with TspRI was performed at 65 °C in 1 × CutSmart Buffer (New England Biolabs). After the digestion, a second PCR was performed using the digested PCR products as templates.

### Immunohistochemistry

CD1a immunostaining was performed using an automatic immunostaining machine HISTOSTAINER 48A (Nichirei Corporation, Japan). The primary antibody was CD1a mouse monoclonal antibody (Leica Biosystem: NCL-CD1a-220) using a 1:50 dilution, and the secondary antibody was Histofine Simple Stain MAX-PO (MULTI) (Nichirei Corporation, Japan). Images of immunostained samples were captured using a NanoZoomer-XR digital scanner (Hamamatsu Photonics). The tumor content was calculated as the ratio of the CD1a (+) area to the whole specimen, using the NDP.analyze U12356 software program.

## Results

### Optimization of TspRI digestion of the BRAF amplicon

**Fig. 1:**
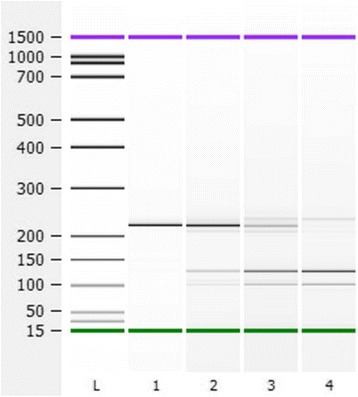
Optimization of TspRI digestion for the BRAF amplicons. 100 ng of *BRAF* PCR fragments amplified from normal DNA were digested with TspRI. Lane 1: No TspRI, 65 °C, 15 min. Lane 2: 5U TspRI, 65 °C, 5 min. Lane 3: 10U TspRI, 65 °C, 10 min. Lane 4: 20U TspRI, 65 °C, 15 min, leading to digestion of the majority of *BRAF* PCR fragments

A 209 bp PCR product including *BRAF* V600E wild-type was amplified from DNA extracted from FFPE blocks of human tonsil tissue (Fig. 1, lane 1). The PCR products were treated with TspRI under various conditions. After digestion at 65 °C for 15 min, the products were almost completely cleaved to give two fragments of 124 and 94 bp (Fig. 1, lane 4).

### Detection of *BRAF* V600E mutations by TspRI digestion

**Fig. 2:**
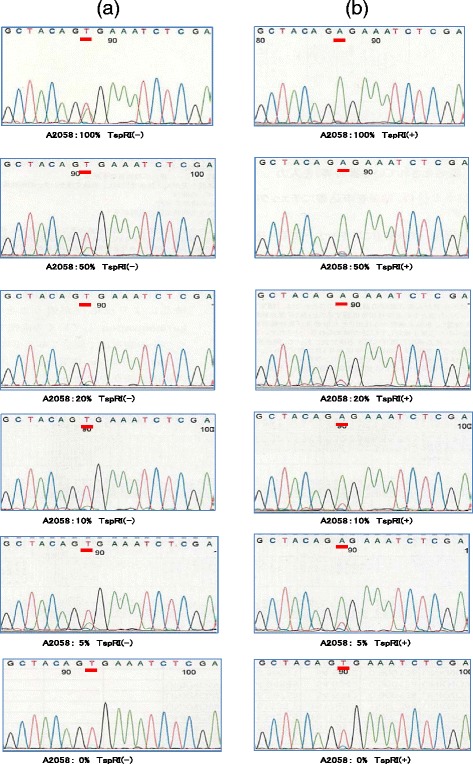
Sequence analysis of samples from six mixtures containing 0–100 % *BRAF* V600Emutation(+) cells. (**a**) Sequences of undigested products, TspRI (-): Where material was derived from 100 % A2058 cells (top panel) the mutant sequence, GAG, contributed approximately 50 % of the total, allowing clear categorization of sample as mutation (+). In all other cases (0–50 %, A2058 cells), it was difficult to judge whether the samples were mutation (+) or (-). (**b**) Sequences of digested products, TspRI (+): All samples containing the mutated (GAG) sequences demonstrated superior amplification of this in comparison to the wild type (GTG) after treatment with TspRI. The negative control containing 0 % A2058 cells (bottom panel) did not show amplification of the mutant sequence. Hence, the *BRAF* mutation could detected in samples containing as little as 5 % of A2058 cells (equating to 2.5 % of total DNA)

Six mixtures of cell lines with (A2058) and without (UE7T-13) the *BRAF* V600E mutation were prepared containing 0, 5, 10, 20, 50 and 100 % of A2058 cells. FFPE cell blocks were prepared from each mixture, and five 10 μm sections of each block were collected in 1.5 ml Eppendorf tubes. Three tubes were prepared for each FFPE blocks to examine in triplicate. DNA was extracted from the samples in each tube for subsequent PCR, TspRI treatment (+ or -), and sequence analysis. The A2058 cell line was heterozygous for the V600E mutation (Fig. 2, A2058 100 %, TspRI (-)), which was thus present in 50 % of *BRAF* sequences derived from this cell line. The sequence analysis of secondary PCR products derived from all proportions of A2058 cells, except 100 % (i.e., 0–50 %), demonstrated that when the primary PCR product template DNA was not treated with TspRI, the wild-type (mutation (-) sequence, GTG(V)), was preferentially amplified compared to the mutation(+) sequence, GAG(E). By contrast, treatment with TspRI prior to secondary PCR led to preferential amplification of the *BRAF* V600E mutation compared to the wild-type sequence, even in the presence of only 5 % of A2058 cells (Fig. 2, A2058 5 %, TspRI (+)). As expected, in the negative control containing UE7T-13 cells alone (A2058: 0 %), TspRI treatment prior to secondary PCR had no effect on the results of the sequencing analysis, indicating that these cells were mutation (-) (Fig. 2, A2058: 0 %, TspRI(+)).

According to these results, we proceeded to analyze 32 FFPE samples from LCH patients as follows: five pieces of 10 μm sections per patient were used for DNA extraction, and 0.25–14 μg of DNA were extracted. DNA (100 ng) from FFPE tissues was used as a template for primary PCR assays. Two 6 μl aliquots of the resulting PCR products were then either treated with TspRI (TspRI(+)) or used as a negative control (TspRI(-)), followed by secondary PCR. The sequences resulting from primary PCR, secondary PCR using undigested template and secondary PCR of TspRI digested template were compared (Table 1).

**Table 1:** The results of sequence analysis for *BRAF* V600 mutations

				Sequence analysis		
Patient NO.	Sex	Age (year)	Tumor content (%)	(A)PCR	(B) PCR+ TspRI(-)	(C) PCR+ TspRI(+)
5	M	1	73.3	(+)	(+)	(+)
13	F	1	73.7	(+)	(-)	(+)
16	M	1	21.1	(+)	(+)	(+)
19	M	2	80.8	(+)	(+)	(+)
32	M	2	90.6	(+)	(+)	(+)
3	M	2	11.8	(+)?	(-)	(+)GAT
6	F	2	58.0	(+)?	(-)	(+)
10	M	5	70.5	(+)?	(-)	(+)
29	M	5	75.6	(+)GAT?	(+)GAT?	(+)GAT
12	F	17	-	(-)	(-)	(+)
15	F	1	31.7	(-)	(-)	(+)
17	M	0	-	(-)	(-)	(+)
20	F	1	12.5	(-)	(-)	(+)
23	M	14	16.3	(-)	(-)	(+)
24	F	6	43.5	(-)	(-)	(+)
25	F	12	43.0	(-)	(-)	(+)
28	F	9	56.1	(-)	(-)	(+)
30	M	2	10.9	(-)	(-)	(+)
31	M	2	24.8	(-)	(-)	(+)
1	F	13	42.5	(-)	(-)	(-)
2	M	1	58.5	(-)	(-)	(-)
4	M	3	53.1	(-)	(-)	(-)
7	F	0	4.3	(-)	(-)	(-)
8	F	4	39.7	(-)	(-)	(-)
9	F	3	27.0	(-)	(-)	(-)
11	F	4	-	(-)	(-)	(-)
14	M	2	100.0	(-)	(-)	(-)
18	F	0	-	(-)	(-)	(-)
21	M	14	48.9	(-)	(-)	(-)
22	F	11	41.0	(-)	(-)	(-)
26	M	12	57.7	(-)	(-)	(-)
27	F	5	83.0	(-)	(-)	(-)

(A) Sequence analysis after primary PCR amplification. Five samples were V600E mutation (+), 23 were mutation (-), and four were ambiguous. (B) Sequence analysis after secondary PCR without TspRI treatment. Four samples were V600E mutation (+), 27 were mutation (-), and one was ambiguous, (C) Sequence analysis after secondary PCR following TspRI digestion. Nineteen samples were mutation (+) and 13 were mutation (-). All four cases that produced ambiguous results after the primary PCR were found to be mutation (+) using our method and ten cases initially classified as mutation (-) were found to be mutation (+). In addition, two mutation (+) samples were shown to have V600D (GTG to GAT) mutations

**Fig. 3:**
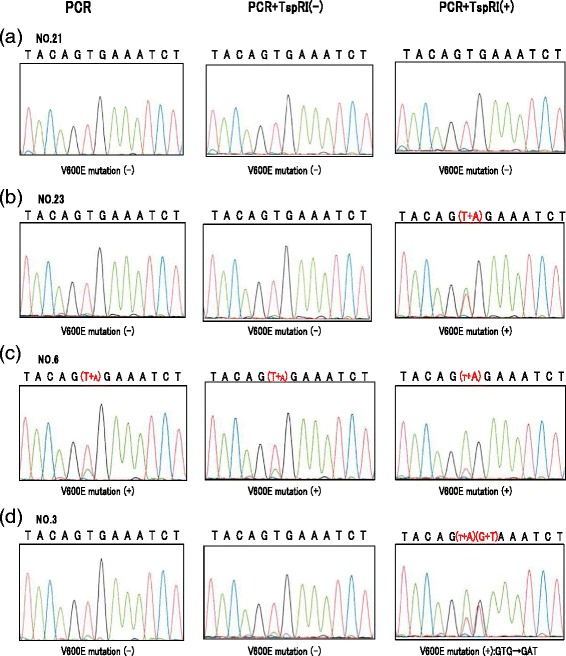
The effect of TspRI restriction enzyme treatment on the *BRAF* sequence analysis of patient samples (**a**) Patient no. 21, representative sample with no V600E mutation, was judged mutation (-):GTG after primary PCR, and this conclusion was not changed by treatment with TspRI followed by secondary PCR; (**b**) patient no. 23 was scored as mutation (-):GTG after primary PCR, but mutation (+):GAG after treatment with TspRI; (**c**) the mutation status of patient no. 6 was difficult to judge from the primary PCR because of overlap between mutation (-):GTG and mutation (+):GAG sequences, however, after treatment with TspRI, the mutation (+):GAG sequence was dominant, indicating a clear mutation (+):GAG status; (**d**) patient no. 3 was initially judged mutation (-):GTG after primary PCR, but mutation (+):GAT status was apparent after treatment with TspRI

From the sequence analysis of the primary PCR products, it was possible to confirm the presence of the *BRAF* V600E mutation in five cases. Twenty-three cases were determined to be mutation (-), and for four cases it was not possible to judge whether or not the mutation was present from the results (Table 1, (A) PCR). In the TspRI (-) group (B), the *BRAF* V600E mutation was detected in four cases, with 27 mutation-negative cases, and one indeterminate result. By contrast, in the TspRI (+) group (C), mutations were detected in 19 cases, including five where the mutation had been detected, four with indeterminate results, and ten cases judged to be mutation (-) by sequencing after only primary PCR. Our results also demonstrate that two of these 19 mutation (+) cases carried a mutation to GAT (D), rather than the more common GAG(E). Four representative examples of the sequence analyses with and without TspRI treatment are presented in Fig. 3.

### Relationship between the tumor content and *BRAF* V600E mutation detection by TspRI digestion and sequencing analysis

**Fig. 4:**
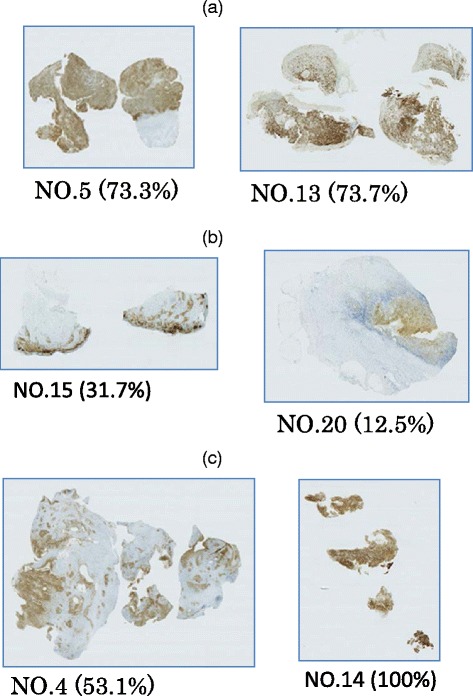
CD1a immunohistochemistry FFPE tissue section from LCH patients stained with CD1a. (**a**) Two samples that were V600E mutation (+) after both primary PCR amplification and TspRI followed by additional PCR amplification. Four of five samples in this category demonstrated up to 70 % tumor content. These cases were easy to detect by primary PCR. (**b**) Examples of staining of samples that were *BRAF* mutation (-) after primary PCR, but mutation (+) after TspRI treatment. Seven of eight such samples contained <50 % tumor content. These samples were not dissected to isolate tumor tissue; nevertheless, a *BRAF* V600E mutation was detected after treatment with TspRI. (**c**) Two samples that were *BRAF* V600E mutation (-) after both primary PCR and after TspRI digestion followed by additional amplification. Tumor content was determined in 11 of 13 samples in this category and ranged from 4.9 to 100 %, hence mutation detection was independent of tumor content

From our experiments using mixtures of cell lines, it was possible to detect the V600E mutation when 5 % of the cells in a mixture were mutation (+). We next examined the relationship between mutation detection by the sequence analysis after TspRI treatment and the ratio of tumor content in patient specimens by immunohistochemical staining. The area of the tumor tissue was calculated as the area of anti-CD1a staining in an FFPE section. Specimens for both immunohistochemical staining and DNA extraction were obtained from the same blocks (Table 1 and Fig. 4).

Four of five cases that were mutation (+) according to the primary PCR analysis had tumor contents of >70 %. Eight cases that changed from mutation (-) after primary PCR to mutation (+) following TspRI treatment had an average tumor content of 29.9 % (minimum content 10.9 %). These results demonstrate that TspRI treatment enables the detection of mutations of *BRAF* V600 (to E or D) when the tumor content of a specimen is low. Two of the four cases with indeterminate results after primary PCR alone had sequence mutations from GTG to GAT, indicating that this change may be difficult to discriminate. Patient nos. 2, 4, 14, 26 and 27 were judged to be mutation (-), despite a high (>50 %) tumor content, indicating a high accuracy of mutation status determination, regardless of the tumor content.

## Discussion

LCH is classified into two categories, single organ and multi-organ involvement. The prognosis is almost good in the case of single organ disease, however the course often leads to a poor prognosis in the case of multi-organ involvement, necessitating chemotherapy. Therefore, the selection of effective therapy is important, since relapse after remission is associated with high mortality [14].

The *BRAF* gene is located on chromosome 7q34, composed of 18 exons, and encodes a mRNA transcript of 2478 bp. *BRAF* genetic missense mutations are associated with the incidence of various types of cancers. *BRAF* mutations have been confirmed, not only in LCH, but also in ECD, melanoma, papillary thyroid cancer, colorectal cancer, HCL and CLL. The V600E mutation accounts for the majority of these mutations, especially in melanoma where approximately 60–70 % cases of melanoma patients have *BRAF* mutations, and about 90 % of these are V600E. Melanoma tumor containing the *BRAF* V600E mutation are resistant to EGFR inhibitors, and treatment with BRAF inhibitors demonstrates remarkable effects on the tumor. Vemurafenib, dabrafenib and trametinib have been used in the treatment of such patients as targeted therapy [15–17]. However, these treatments are not effective in patients without *BRAF* mutations. Immunotherapy with ipilimumab, which targets tumors without mutant *BRAF*, is appropriate for these patients [18]. Hence, sensitive methods for the detection of *BRAF* genetic mutations are important for the selection of effective therapies and in the diagnosis. Generally, tumor tissue biopsies from LCH patients include some normal tissue and many inflammatory cells. When analyzing the DNA sequences from such samples, the proportion of tumor cells is an important issue that can affect the analysis and correct judgment of the mutation status.

To address this problem, we verified the accuracy of our new method for mutation detection by comparing sequences of the *BRAF* V600E mutation amplified by the popular method of PCR alone, with those generated by our method combining PCR with TspRI restriction enzyme digestion. A total of 32 tissue samples from LCH patients were screened for the presence of *BRAF* V600E mutation. Based on the findings of conventional PCR and a sequence analysis, we confirmed only five cases of *BRAF* V600E mutation, with four cases giving ambiguous results. Treatment with TspRI reduced the amount of DNA template from wild-type tissue; consequently, we were able to detect 19 cases with clear mutations, with four cases where a clear result could not be obtained. In addition, we were able to detect not only the V600E mutation, but also V600D. The detection of 19 *BRAF* V600 (E or D) mutations from 32 patients, corresponds to 59 % of all cases in this series. This incidence is similar to that found by another study (57 %), which used iPLEX chemistry methodology [19, 20]. In addition, our results from DNA samples derived from a *BRAF* V600E heterozygous mutant cell line mixed with a wild-type cell line indicate that the mutation can be detected when only 5 % of cells are from the mutation (+) cell line, equal to 2.5 % of all cells.

For the *BRAF* V600E mutation analysis, we assessed several methods for DNA extraction from the FFPE samples. However, some methods were limited to only samples with more than 50 % of tumor tissue, or the necessity for microdissection of the tumor tissue [5, 6]. These requirements can preclude an analysis due to the tumor size or condition, or require specialists who are able to discriminate tumor tissue, and are therefore likely to be performed inconsistently in different laboratories. Furthermore, these methods require specialized and expensive equipment, which will limit analyses to those laboratories with access to such facilities. The method we herein propose only requires access to a regular thermal cycler and sequencer and does not require difficult pathology methods of tissue selection by microdissection. As it is now common for commercial companies to provide sequencing services, it will be possible to use this method for the diagnosis, not only in specialized facilities, but also in general clinical laboratories.

## Conclusion

In conclusion, we herein presented a new and highly sensitive method to detect *BRAF* V600 mutations. This screening assay is anticipated to improve the efficiency of cancer therapy by identifying patients who required BRAF V600 inhibitors to preserve EGFR-mediated responses. The screening method presented in this report is expected to play an important role in reducing unnecessary treatments and thus making it easier to select the most effective therapies.
